# Triacylglycerols: Fuelling the Hibernating *Mycobacterium tuberculosis*

**DOI:** 10.3389/fcimb.2018.00450

**Published:** 2019-01-09

**Authors:** Rahul Kumar Maurya, Suman Bharti, Manju Y. Krishnan

**Affiliations:** Microbiology Division, CSIR-Central Drug Research Institute, Lucknow, India

**Keywords:** *Mycobacterium*, persistence, lipid inclusions, triacylglycerol, lipid bodies

## Abstract

*Mycobacterium tuberculosis* (*Mtb*) has the remarkable ability to persist with a modified metabolic status and phenotypic drug tolerance for long periods in the host without producing symptoms of active tuberculosis. These persisters may reactivate to cause active disease when the immune system becomes disrupted or compromised. Thus, the infected hosts with the persisters serve as natural reservoir of the deadly pathogen. Understanding the host and bacterial factors contributing to *Mtb* persistence is important to devise strategies to tackle the *Mtb* persisters. Host lipids act as the major source of carbon and energy for *Mtb*. Fatty acids derived from the host cells are converted to triacylglycerols (triglycerides or TAG) and stored in the bacterial cytoplasm. TAG serves as a dependable, long-term energy source of lesser molecular mass than other storage molecules like glycogen. TAG are found in substantial amounts in the mycobacterial cell wall. This review discusses the production, accumulation and possible roles of TAG in mycobacteria, pointing out the aspects that remain to be explored. Finally, the essentiality of TAG synthesis for *Mtb* is discussed with implications for identification of intervention strategies.

## Introduction

A recent global tuberculosis (TB) report by the World Health Organization reveals that the TB epidemic is larger than previously estimated despite a global fall in the number of TB deaths and the TB incidence rate [World Health Organization (WHO), 2016]. Also, “The End TB strategy”approved in 2014 calls for a 90% reduction in TB deaths and 80% reduction in TB incidence rates by the year 2030, compared to 2015. The objectives of current anti-tuberculosis therapy are to (1) decrease the severity of the disease by rapidly reducing the number of actively growing *Mycobacterium tuberculosis* (*Mtb*) in the patient, thereby, preventing death and halting bacterial transmission (2) prevent relapse by eliminating all populations of persisting bacilli; and (3) prevent development of drug resistance (Nahid et al., 2016). Persistence of *Mtb* is often described as a condition in which multiple growth limiting factors within the host tissues force a certain population of the bacilli to undergo a state of suboptimal growth or complete growth arrest accompanied by phenotypic drug tolerance (Gold and Nathan, 2017). Persisters exhibit a modified metabolic status in which aerobic respiration and ribosomal function are decreased while lipid utilization is increased (Garton et al., 2008). Persisters, under circumstances of dysregulated immunity may lead to reactivation TB. In fact, the lengthy treatment regimen for TB is necessary to eliminate the persisting population of *Mtb*. Hence understanding how *Mtb* persists in the face of adverse conditions in the host is crucial to devise strategies to reduce the duration of treatment and minimize post-treatment relapse.

### *M. tuberculosis* Accumulating Triacylglycerols Is Slow/Non-replicating and Drug Tolerant

Triacylglycerol (TAG), a triester of glycerol with fatty acids, is the major energy depot of all eukaryotes and bacteria of the actinomycetes group. Actinomycetes that accumulate large amounts of TAG include *Mycobacterium, Rhodococcus, Nocardia*, and *Streptomyces* (Olukoshi and Packter, 1994; Alvarez et al., 2000; Alvarez and Steinbüchel, 2002). The energy derived from the complete oxidation of the fatty acyl chains of TAG is estimated to be more than twice the same weight of carbohydrate or protein (Sturley and Hussain, 2012). Being nonpolar, triacylglycerols can store nearly six times more energy than the same amount of hydrated glycogen and also provide energy for longer time than the fast catabolising glycogen (Berg et al., 2002).

**Figure 1:**
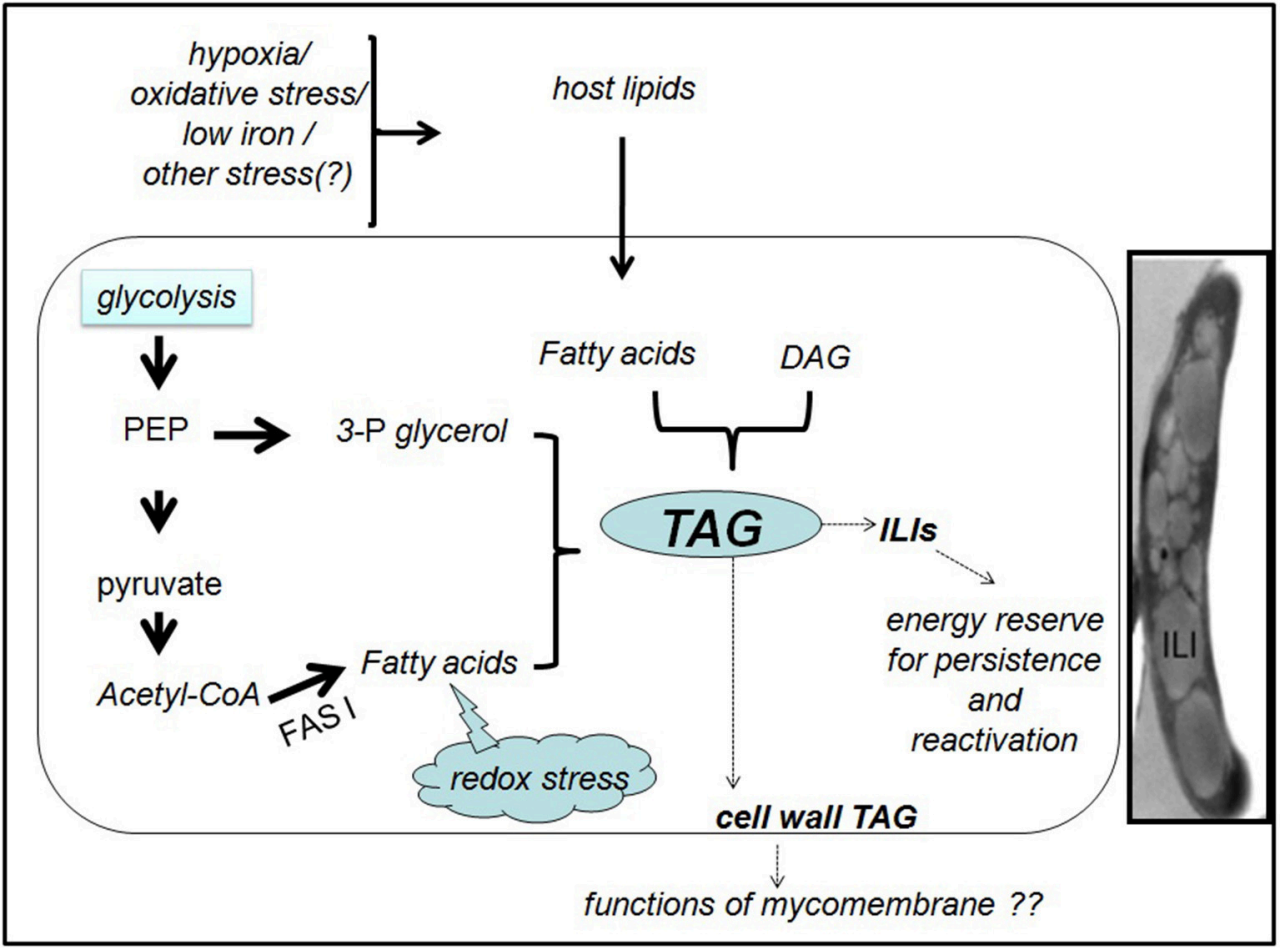
Generation and fate of TAG in *M. tuberculosis* inside the host. In presence of a lipid-rich diet and/ or various indicated stresses, *M. tuberculosis* synthesizes TAG, either *de novo* from fatty acids and 3-phosphoglycerol (3-P glycerol) or from pre-existing DAG. Phosphoenol pyruvate (PEP) and pyruvate may provide the precursors for *de novo* TAG synthesis. Reductive stress generated due to excess metabolic degradation of fatty acids may also induce the conversion of excess fatty acids to TAG. The cytoplasmic TAG in the ILIs may promote the pathogen survival during dormancy and reactivation. Functions of the cell wall associated TAG are unknown. Inset: Transmission electron microscopy image of *Mtb* in TB patient sputum showing the intracellular lipid inclusion (ILI)s. The image is reproduced with permission from Vijay et al. (2017).

Evidences suggest that *Mtb* depends on host nutrients especially lipids to survive in the host (Bloch and Segal, 1956). Intracellular inclusions were detected in mycobacterial species by several studies since 1946 (reviewed in Garton et al., 2002). The use of fluorescent lipid probes showed presence of intracellular lipid inclusions (ILIs) in *M. smegmatis* and *Mtb* (Christensen et al., 1999; Garton et al., 2002). Thin layer chromatography, NMR and GC-MS analyses of nonpolar lipid extracts from the ILI-rich *M. smegmatis* cultures showed TAG containing variable fatty acids as the primary lipid component of ILIs (Garton et al., 2002). The term ILIs is used in this article to describe the lipid bodies in the mycobacterial cytoplasm. *Mtb* with ILIs has been detected in patient sputa as well as in *in vitro* cultures modeling non-replicating persistence (Garton et al., 2008; Rodríguez et al., 2014; Vijay et al., 2017) (Figure 1, inset). Interestingly, it has been observed that the ILI-positive bacilli counts correlated significantly with time to detect *Mtb* growth in diagnostic liquid cultures, which indicated that being non-replicating, bacilli with ILIs took more time to initiate growth (Garton et al., 2008). It is important to note that various *in vitro* studies have shown that *Mtb* and other mycobacteria exhibit phenotypic drug tolerance subsequent to accumulation of ILIs (Deb et al., 2009; Rodríguez et al., 2014; Hammond et al., 2015). A *Mtb* H37Rv mutant with reduced TAG synthesis has been found to be more sensitive to antibiotics during mice infection than the wild type and complemented mutant (Baek et al., 2011).

## Biosynthesis of TAG in Mycobacteria

### Potential Pathways Leading to TAG Biosynthesis

Garton et al demonstrated that *M. smegmatis* made ILIs during stationary phase from simple carbon sources in low-nitrogen conditions, but the formation was rapid if fatty acids were provided (Garton et al., 2002). Transcript analysis suggests that *Mtb* entering growth arrest due to *in vitro* or *in vivo* stress undergo metabolic adaptation for long-term persistence (Shi et al., 2010). Shi et al., induced *in vitro* growth arrest in *Mtb* either by hypoxia or by nitric oxide treatment while culturing in standard media containing fatty acids or media with defined carbon sources but no fatty acids. The transcript data along with flux balance analysis using an *in silico* metabolic network proposed a metabolic model for *Mtb* persisters where carbon flow is redistributed from providing energy and biosynthetic precursors for bacterial growth to accumulating storage compounds like TAG and glutamate (Shi et al., 2010). According to this model, in non replicating *Mtb*, carbon is rerouted to TAG synthesis in at least two ways. First, due to the downregulation of glycolysis, TCA cycle and pentose phosphate pathway, pyruvate and phosphoenol pyruvate are preferentially used for glyceroneogenesis and TAG synthesis. Second, uncoupling of FASI and FASII (fatty acid synthase I and II systems sequentially involved in mycobacterial fatty acid biosynthesis), FASI products are rerouted to TAG synthesis. An extensive system biology study of dormant *Mtb* under *in vitro* hypoxia proposed that metabolites upstream of diacylglycerol (DAG) decrease in production while TAG accumulation may result from conversion of existing DAGs to TAGs by triacylglyceride synthases (Galagan et al., 2013). Figure 1 summarizes the potential routes of TAG synthesis in *Mtb*.

In more direct experimental approaches, pulsing the macrophages with fluorescent labeled fatty acid has shown that intracellular *Mtb* imports fatty acids from the macrophage to synthesize its own TAG (Daniel et al., 2011; Nazarova et al., 2017). Fatty acid uptake by *Mtb* takes place through the Mce1 transporter complex (Nazarova et al., 2017). While *Mtb* has been shown to derive fatty acids from TAG accumulating inside lipid loaded macrophages (Daniel et al., 2011), a recent study shows that the bacilli were able to accumulate ILIs even in the absence of macrophage TAG and hence suggests that host TAG may not be the primary source of fatty acids for *Mtb* (Knight et al., 2018).

### Molecular Variety of TAG in Mycobacteria

Detection of TAG species showing variable TLC mobilities in *M. smegmatis* suggests the ability of the mycobacteria to synthesize a large variety of TAG (Garton et al., 2002). In addition to the conventional TAG, pathogenic as well as non-pathogenic mycobacteria contain unusual TAGs bearing a meromycolate substituent called monomeromycolyl-diacylglycerol or MMDAG (Kremer et al., 2005). Mass spectrometry analysis of *M. smegmatis* biofilm revealed a complex mixture of TAG and MMDAG (Purdy et al., 2013). Various species of TAG and MMDAG consisted of the common structure in which Δ^9^18:1- and 16:0-fatty acyl substituents are exclusively located at sn-1 and sn-2, respectively. The sn-3 position of TAGs contained saturated or mono-unsaturated fatty acyl chains of chain lengths varying from C10 to C26. MMDAG species varied with respect to the meromycolylic fatty acyl chain (C43-C57) at sn-3 (Purdy et al., 2013), Eventhough the structures of *Mtb* TAGs and MMDAGs are not yet known, at least 32 species of TAG have been detected by mass spectrometry in *Mtb*H37Rv (Martinot et al., 2016).

### TAG Synthases of Mycobacteria

Experimental evidence of TAG synthesis in mycobacteria came from a study in which *M. avium* incorporated 14-C-palmitic acid into TAG within minutes of exposure to the fatty acid (McCarthy, 1971). Bacteria pulsed with 14-C-palmitic acid for 30 min released higher levels of 14-CO_2_, when supplemented with both glycerol and unlabelled palmitic acid rather than palmitic acid alone, demonstrating the utilization of the newly synthesized TAG. Subsequently, diglyceride acyltransferase [EC 2.3.1.20] (triacylglycerol synthase) activity was detected in a cell-free extract from *M. smegmatis* (Nakagawa et al., 1976). This class of enzymes are solely responsible for the final acylation of diacylglycerols (DAG).

Daniel et al identified 15 genes in *Mtb* H37Rv genome, which code for putative triacylglycerol synthase (Tgs)s (Daniel et al., 2004). When expressed in the *E. coli* host, all the 15 gene products showed Tgs activity with diolein and oleoyl-CoA as substrates. While Rv3130c (tgs1) showed the highest activity among all, the remaining proteins showed moderate to very low activity with diolein and oleoyl-CoA. Some of the proteins also showed mild wax ester synthase activity, which was however not correlated to their Tgs activity (Daniel et al., 2004). In *Mtb*, the expression of some of these genes, particularly *Rv3130c*/*tgs1*, was up-regulated under hypoxia/nitric oxide –induced *in vitro* non-replicating persistence, along with a corresponding increase in TAG accumulation. In addition to these putative Tgs, TAGs in *Mtb* could also be synthesized by the mycolyl transferase Ag85A (Rv3804c) by transesterification of DAG (Elamin et al., 2011). Table 1 lists the confirmed and putative TAG synthases in *Mtb* along with some basic information. The Tgs members may differ from each other with respect to substrate specificity and inducing microenvironment.

**Table 1:** TAG synthases in *M.tuberculosis* H37Rv.

**Sl No**	**Confirmed /putative TAG synthase in Mtb H37Rv**	**Essentiality for growth^**a,b,c**^**	**Presence of orthologs in mycobacterial sp**.	**Up-regulation during stress and/or growth arrest**	**Subcellular localization^**i**^**
1	Rv0221	No^a,b,c^	Pathogenic and non-pathogenic	No	Cell membrane fraction
2	Rv0895	No^a,c^ Yes^b^	Pathogenic	No	Cellwall fraction
3	Rv1425	No^a,b,c^	Pathogenic	No	Cell membrane fraction
4	Rv1760	No^a,b,c^	Pathogenic	No	Not known
5	Rv2285	No^a,b,c^	Pathogenic	No	cell membrane fraction
6	Rv2484c	No^a,b,c^	Pathogenic and non-pathogenic	No	Cell membrane fraction
7	Rv3087	No^a,b^ Yes^c^	Pathogenic and non-pathogenic	No	Cell membrane fraction
8	Rv3088 (Tgs4)	No^a,b,c^	Pathogenic	Low pH *invitro*^d^	Not known
9	Rv3130c (Tgs1)	No^a,b,c^	Pathogenic and non-pathogenic	*In vitro* hypoxia^e^, nitrosative stress^e^, infected adipocyte model^f^, sputum bacilli^g^	Cellwall & cell membrane fractions
10	Rv3233c	No^a,b,c^	Pathogenic	No	Not known
11	Rv3234c (Tgs3)	No^a,b,c^	Pathogenic	*In vitro* hypoxia^e^, infected adipocyte model^f^	Not known
12	Rv3371	No^a,b^ Yes^c^	Pathogenic	*In vitro* hypoxia^e^, nitrosative stress^e^, infected adipocyte model^f^	Cell membrane fraction
13	Rv3480c	No^a,c^ Yes^b^	Pathogenic	No	Not known
14	Rv3734c (Tgs2)	No^a,b,c^	Pathogenic and non-pathogenic	*In vitro* hypoxia^e^, nitrosative stress^e^, infected adipocyte model^f^, nutrient starvation^h^	Cell membrane fraction
15	Rv3740c	No^a,b,c^	Pathogenic	No	
16	Rv3804c (FbpA)	Not clear	Pathogenic and non-pathogenic	Infected adipocyte model^f^	Secreted

a *Essential for in vitro growth (Sassetti et al., 2003; Griffin et al., 2011; DeJesus et al., 2017*.

b *Essential for survival in primary murine macrophages (Rengarajan et al., 2005)*.

c *Essential for survival in mouse spleens (Sassetti and Rubin, 2003)*.

d *(Singh et al., 2005)*;

e *(Daniel et al., 2004)*;

f *(Rastogi et al., 2016)*.

g *(Garton et al., 2008)*;

h *(Betts et al., 2002)*;

i *(Mawuenyega et al., 2005)*.

Mutagenesis studies suggest that none of the confirmed/putative Tgs are essential for *in vitro* growth of *Mtb* H37Rv (Sassetti et al., 2001; Griffin et al., 2011; DeJesus et al., 2017). Since H37Rv strain does not accumulate TAG during *in vitro* growth unlike the clinical *Mtb* isolates, the *tgs* gene essentiality data for H37Rv cannot be extended to clinical strains. *tgs1* disruption inhibits most of the TAG accumulation under acidic, static or hypoxic growth conditions *in vitro* (Sirakova et al., 2006). *tgs1* deletion mutant was also found unable to arrest its growth under *in vitro* hypoxia, low pH and low iron (Baek et al., 2011). TAG synthesis was drastically reduced in a *tgs1-* deletion mutant of *Mtb* inside foamy macrophages (Daniel et al., 2011). *Mtb*- Tgs 1 preferably esterifies C26:0 fatty acyl chains to DAG (Sirakova et al., 2006). The residual TAG detected in the *tgs1*deletion mutant did not have C26:0 fatty acyl chains, indicating the substrate specificity of the multiple Tgs. A Tgs of *M. abscessus* with reasonable homology to *Mtb*Tgs 1, has been reported to be responsible for most of the TAG synthesized during exponential phase in *M. abscessus* (Viljoen et al., 2016).

Two of the putative Tgs- Rv3087 and Rv3371 were found essential for *in vivo* growth of H37Rv in C57BL/6J mouse spleen, by transposon site hybridization (Sassetti and Rubin, 2003). We have reported that an unmarked deletion mutant of *Rv3371* continued replication and thus failed to enter persistence under *in vitro* hypoxia, low iron or nitrosative stress (Rastogi et al., 2017). Moreover, transposon mutants of *tgs1* and *Rv3371* were among the “over-represented” mutants under low oxygen environment, which suggested their inability to arrest cell division (Baek et al., 2011). Rv3371 has the closest aminoacid sequence (43%) similarity to Tgs1 than any other Tgs in *Mtb*H37Rv (Daniel et al., 2004). Two other putative Tgs- Rv0895 and Rv3480c were found essential for the survival of H37Rv in primary murine macrophages, by transposon site hybridization (Rengarajan et al., 2005). We used an adipocyte-*Mtb* infection model to mimic the nutritional status inside foamy macrophages.Transcript analysis by quantitative RT-PCR showed that *tgs1, tgs2, Rv3371*, and *Rv3804c* are the predominant *tgs* genes upregulated in *Mtb* H37Rv during survival inside adipocytes (Rastogi et al., 2016). All these studies point out that expression of *tgs* genes is regulated and their expression is induced or down regulated by specific signals. Hence, validation of individual Tgs proteins as drug target requires testing of their essentiality for *in vivo* growth or persistence.

### TAG Utilization by Mycobacteria

TAGs are hydrolysed to provide free fatty acids to enter β-oxidation, which in turn can provide energy and acetyl Co-A. This acetyl Co-A is thought to be used for both lipid synthesis via FAS I and anapleurosis of the TCA cycle (Shi et al., 2010). There are at least 24 genes in the *Mtb* H37Rv genome that code for lipase/esterase proteins (Deb et al., 2006). Of these only one protein Rv3097c (LipY), a hormone-sensitive lipase was found to hydrolyze TAG with long chain fatty acids. A lipY- deficient *Mtb* H37Rv mutant, showed drastically decreased TAG utilization under nutrient deprived condition (Deb et al., 2006). A recent study using BCG infected foamy macrophages revealed that LipY functions in two forms- as an extracellular lipase that hydrolyses the host TAG and as a cytoplasmic lipase that hydrolyses the TAG in bacterial ILIs (Santucci et al., 2018). This study not only supports the earlier studies that LipY is the major TAG lipase of *Mtb*, but also suggests the additional involvement of other mycobacterial lipases in ILI formation and utilization. We used 3T3L1 adipocytes as an alternative to faomy macrophages and found intra-adipocyte *Mtb* to significantly upregulate the expression of *lipY* and a few other *lip* genes (Rastogi et al., 2016).

### Location of TAG in Mycobacteria

TAG is accumulated in mycobacteria as two forms- peripheral deposits associated with the cell envelope and inclusion bodies in the cytoplasm (ILIs) (Christensen et al., 1999). DAGs and TAGs have been found in substantial amounts in the the outer membrane (mycomembrane) of *M. smegmatis* (Bansal-Mutalik and Nikaido, 2014). Much earlier, Ortalo-Magne'et al detected TAGs deep inside *Mtb* capsule and also surface-exposed TAG in *M. smegmatis, M. avium*, and *M. aurum* (Ortalo-Magné et al., 1996). TAG production predominantly through a Tgs1 homolog, during exponential phase has also been reported for *M. abscessus*. Interestingly, electron microscopy images revealed that there was no accumulation of ILIs, which suggests the possible accumulation of TAG in the cell wall (Viljoen et al., 2016).

In *Mtb*, an efflux pump Rv1410 and lipoprotein LprG which are encoded by the *Rv1410c-Rv1411c* operon, were found to transport TAG from cytoplasm to the outermembrane. Over expression of these proteins in *Mtb* also led to release of TAG into the culture medium (Martinot et al., 2016). It was observed that LprG-Rv1410 were important to protect the bacilli from toxic byproducts of cholesterol through their disposal *via* TAG from the cytoplasm. It remains to be seen if the export of TAG from cytoplasm is just a regulatory mechanism of intracellular TAG accumulation or the cell wall associated TAG has other unknown functions. The observation in *M. abscessus* of increased TAG synthesis without corresponding formation of ILIs (Viljoen et al., 2016) suggests that accumulation of TAG in the cell wall is not to get rid of the excess cytoplasmic TAG.

### Structure and Formation of ILIs in Mycobacteria

Lipid bodies in eukaryotes consist of a hydrophobic core of neutral lipids, mostly TAGs and steryl esters, or wax esters surrounded by a phospholipid (PL) hemimembrane with a few proteins bound to the surface of the particles (Murphy, 2001; Tauchi-Sato et al., 2002). A few proteins have been identified to be associated to ILIs in mycobacteria. They include TAG synthesizing and degrading enzymes (Low et al., 2010) and stress responsive proteins (Low et al., 2010; Armstrong et al., 2016). Proteins with amphipathic helix motifs are thought to associate with ILIs in mycobacteria (Armstrong et al., 2018). Low et al isolated ILI- associated proteins from dormant *M. bovis* BCG in hypoxic culture and identified BCG1721, tgs1, tgs2, BCG1489c, BCG1169c, and hspX (α-crystallin) (Low et al., 2010). The proteome of ILIs may differ with the condition that induces their formation. MPER-1 is a protein in *Mtb* that has been identified to be essential for TAG accumulation in ILIs during dormancy induced by a combination of *in vitro* stresses (Daniel et al., 2016). This protein has weak amino acid similarity to mammalian perilipin1, which is exclusively associated to lipid droplets in adipocytes and regulates lipolysis (Brasaemle et al., 2009).

TAG and other neutral lipids in eukaryotes are synthesized in the lumen of endoplasmic reticulum (ER) and are rapidly deposited into cytoplasmic lipid droplets that bud out of the ER (Murphy and Vance, 1999; Sturley and Hussain, 2012). Murphy and Vance proposed the plasma membrane as the site of origin of ILIs in bacteria (Murphy and Vance, 1999). Later, studies on *Acinetobacter calcoaceticus* and *Rhodococcus opacus* by Wältermann et al demonstrated a model for ILI formation in bacteria (Wältermann et al., 2005). According to this model, wax esters and TAG are synthesized at the bacterial plasma membrane by respective enzymes which are docked to the membrane. The newly synthesized lipids form small lipid droplets get coated by a monolayer of phospholipids. Small lipid droplets subsequently conglomerate to membrane-bound lipid-prebodies which are then released into the cytoplasm. The lipid prebodies mature as the small lipid droplets inside them coalesce.

Most of the Tgs proteins of *Mtb* H37Rv have been detected in the cell membrane fraction (Table 1). Likewise, the Tgs proteins of *M. abscessus* have been found exclusively in cell wall and cell membrane fractions (Viljoen et al., 2016). In addition to the predominant presence in the cell membrane fraction, minor amounts of Tgs proteins have been found in cytosolic fraction (Stöveken et al., 2005; Rastogi et al., 2017). Since these proteins do not have transmembrane domain, their membrane localization is thought to be mediated by ionic or hydrophobic interactions.

### Factors That Induce Formation of ILIs in Mycobacteria

TAG accumulation during exponential growth has been reported in hypervirulent *Mtb* strains of the W-Beijing Lineage (Reed et al., 2007). The study also showed that Rv3130c/tgs1, which belongs to the DosR regulon, was responsible for the TAG synthesis. Since all the DosR controlled genes were up-regulated, it was presumed that it “pre- adaptated” these hyper virulent strains to the stress-filled microenvironments *in vivo*. However, this study used only lipid analysis by TLC and did not do ultrastructural study to confirm the accumulation of ILIs. Accumulation of ILIs during exponential growth *in vitro* by clinical isolates of *Mtb*, but not by laboratory adapted strain H37Rv, has been reported recently (Vijay et al., 2017). Hence, it can be speculated that stress encountered *in vivo* might stimulate TAG accumulation in *Mtb*. The same strains when grown *in vitro* under optimal conditions may initially retain the property, but gradually lose it as they get adapted to the *in vitro* growth. Thereafter, TAG accumulation might be induced only under stress conditions, including stationary phase (Hammond et al., 2015).

#### Fatty Acids

Lipid loaded macrophages known as foamy macrophages in the tubercular granuloma play a key role in persistence of pathogenic mycobacteria (Peyron et al., 2008; Cáceres et al., 2009; Russell et al., 2009; Caire-Brändli et al., 2014). Eventhough hypoxic culture conditions greatly enhance the accumulation of TAG containing lipid bodies by infected macrophages, macrophages under normoxic conditions also accumulate lipid bodies (Daniel et al., 2011). *M. marinum* inside the host *Dictyostelium* acquires host fatty acids after the host lipid bodies fuse with the *Mycobacterium-*containing vesicle (Barisch et al., 2015; Barisch and Soldati, 2017). *Mtb* inside adipocytes also accumulate ILIs (Neyrolles et al., 2006; Agarwal et al., 2014). *M. canettii* has been shown to replicate and accumulate ILIs inside brown and white preadipocytes (Bouzid et al., 2017). However, a more recent study demonstrated that macrophage lipid body formation during *Mtb* infection is part of an adaptive immune response activated by IFN-γ (Knight et al., 2018). The study also found that in the absence of macrophage lipid bodies the bacilli were able to accumulate ILIs, but the presence of lipid bodies induced by IFN-γ impaired the accumulation.

*Mtb* grown in presence of long chain fatty acids rather than dextrose as sole carbon source accumulated ILIs, while acquiring a slowed-growth and drug-tolerant phenotype (Rodríguez et al., 2014). Too much metabolic degradation of fatty acids can increase the cytoplasmic pool of reducing equivalents leading to a redox imbalance. Thus, the reductive stress is thought to activate conversion of excess free fatty acids to TAG and complex lipids.

#### Hypoxia and Other Stresses

Both laboratory and clinical strains of *Mtb*, when exposed to *in vitro* hypoxic condition were found to accumulate ILIs (Garton et al., 2008). ILIs were observed in bacilli in both NRP (non-replicating persistence) 1 and NRP 2 stages corresponding to 1 and 0.06 % oxygen saturation, respectively. *M. bovis* BCG accumulates TAG during entry into hypoxia-induced dormancy and enhances its TAG hydrolytic activity up on exit from dormancy, suggesting the significance of TAG for dormancy and reactivation of mycobacteria (Low et al., 2009).

*Mtb*, when subjected to combined stresses *in vitro* consisting of low oxygen, high CO_2_, low nutrient and acidic pH, accumulated TAG and wax ester (Deb et al., 2009). The bacilli also stopped replicating, lost acid-fastness, and showed phenotypic drug tolerance. Oxidative stress by H_2_O_2_, and sublethal concentrations of isoniazid in the culture medium induced ILIs in clinical *Mtb* strains but not in the laboratory strain H37Rv. Too low iron in the medium induced ILIs in clinical strains as well as H37Rv (Vijay et al., 2017). Iron depletion in the medium led to increased abundance of DAG, TAG and wax esters in *Mtb* (Bacon et al., 2007). Mycobactin containing microvescicles secreted by *Mtb* grown in low iron medium were enriched in DAG and TAG as well as phosphatidylethanolamines (Prados-Rosales et al., 2014). We have reported decreased release of nile red staining microvesicles from a deletion mutant of *Rv3371*, a possible *tgs*, grown in low iron medium (Rastogi et al., 2017). An increased accumulation of DAG and TAG in the mycomembrane could occur prior to budding of microvesicles, which are induced by low iron and other yet unidentified conditions. Actual role of these acylglycerols in the mycomembrane is worth investigating.

Potential factors inducing TAG synthesis, possible pathways leading to TAG formation and the fate of TAG in mycobacteria are summarized in Figure 1.

### Implications of Inhibiting TAG Synthesis in Pathogenic Mycobacteria

TAG synthesis is likely important to *Mtb* in at least three ways: (1) storage of carbon and energy for use during persistence and reactivation (Low et al., 2009; Galagan et al., 2013), (2) reduce the toxic burden of free fatty acids causing the reductive stress (McCarthy, 1971; Weir et al., 1972), and (3) reduce the carbon flux through tricarboxylic acid cycle and thereby arrest growth and acquire antibiotic tolerance (Baek et al., 2011). Hence ideally inhibiting TAG synthesis should interfere with dormancy, persistence, survival in a lipid-rich microenvironment, and antibiotic tolerance. Another unexplored role of TAG for mycobacteria is linked to its presence in the mycomembrane (Ortalo-Magné et al., 1996; Bansal-Mutalik and Nikaido, 2014). Interrupting TAG transport from cytoplasm to mycomembrane led to reduced virulence of the *Mtb* strain in mouse (Martinot et al., 2016).

## Conclusions

Triacylglycerols are associated with the long-term persistence of *Mtb*. Evidences suggest that they are an important source of energy for the persisting, slow metabolizing population of the bacilli in the host (Low et al., 2009; Shi et al., 2010; Galagan et al., 2013). The presence of TAG in the mycomembrane is intriguing, especially in the absence of ILIs and indicates additional roles for TAG in the pathogen. Although there are multiple genes coding for triacylglycerol synthases in the *Mtb* genome, only one (*tgs1*) has been explored so far. It is interesting to note that the members of this family are differentially regulated at the transcription level and also the fact that some of the members are exclusively present in pathogenic mycobacteria. Presence of more than a dozen Tgs, the extreme diversity of TAG species synthesized by them, their differential expression induced by multiple stress conditions and the TAG distribution between cell envelope and cytoplasm urge for investigations on the various members of the *Mtb-*Tgs family. The proteome of ILIs may be dynamic, changing with the condition inducing ILIs and is likely to include proteins that regulate the TAG content.

## Author Contributions

RKM, SB, and MK collected the references, prepared the figure and wrote the manuscript.

### Conflict of Interest Statement

The authors declare that the research was conducted in the absence of any commercial or financial relationships that could be construed as a potential conflict of interest.
